# Inhibition of DDAH1, but not DDAH2, results in apoptosis of a human trophoblast cell line in response to TRAIL

**DOI:** 10.1093/humrep/dev138

**Published:** 2015-06-16

**Authors:** B.A. Lumicisi, J.E. Cartwright, K. Leslie, A.E. Wallace, G.S. Whitley

**Affiliations:** Reproductive andCardiovascular Research Group, Institute of Cardiovascular and Cell Sciences, St George's University of London, Cranmer Terrace, London SW17 0RE, UK

**Keywords:** extravillous trophoblast, apoptosis, TRAIL, DDAH, ADMA

## Abstract

**STUDY QUESTION:**

Does inhibition of dimethylarginine dimethylaminohydrolase (DDAH) increase the sensitivity of trophoblasts to TRAIL-induced apoptosis?

**SUMMARY ANSWER:**

Inhibition of DDAH1, but not DDAH2, increases the sensitivity of trophoblasts to TRAIL-induced apoptosis.

**WHAT IS KNOWN ALREADY:**

Successful human pregnancy is dependent on adequate trophoblast invasion and remodelling of the maternal spiral arteries. Increased trophoblast apoptosis is seen in pregnancies complicated by pre-eclampsia. The mechanism underlying this increase is unknown. We have previously shown that nitric oxide (NO) is involved in regulating trophoblast motility and invasion, and have also demonstrated an important role for NO in regulating trophoblast sensitivity to apoptotic stimuli. DDAH is an enzyme that metabolizes asymmetric dimethylarginine (ADMA), an endogenous inhibitor of NO synthesis, previously shown to be elevated in the plasma of pre-eclamptic mothers.

**STUDY DESIGN, SIZE, DURATION:**

This study used the human extravillous trophoblast-derived cell line SGHPL-4 cells. All experiments were performed at least three times.

**PARTICIPANTS/MATERIALS, SETTING, METHODS:**

The effect of DDAH on trophoblast apoptosis was examined using siRNA and time-lapse microscopy. Changes in the expression of DDAH were followed by PCR and western blot analysis. Receptor expression was followed by flow cytometry.

**MAIN RESULTS AND THE ROLE OF CHANCE:**

Inhibiting the expression of DDAH1, but not DDAH2, resulted in a significant increase in the sensitivity of the EVT cell line SGHPL-4 to tumour necrosis factor related apoptosis inducing ligand (TRAIL) induced apoptosis (*P* < 0.01). This response could be mimicked by the addition of Asymmetric Dimethylarginine (ADMA), an endogenous inhibitor of NO synthesis and the substrate for both isoforms of DDAH. We further showed that this increased sensitivity to apoptosis is accompanied by a significant increase in the expression of TRAIL receptor 2 (TR2; *P* < 0.05) but not TRAIL receptor 1 (TR1).

**LIMITATIONS, REASONS FOR CAUTION:**

This study was performed only *in vitro* using a well characterized trophoblast cell line, SGHPL-4, derived from first trimester extravillous trophoblasts.

**WIDER IMPLICATIONS OF THE FINDINGS:**

This study provides new insight into the role of the DDAH/ADMA pathway in the regulation of trophoblast function. Both dysregulation of DDAH and the accumulation of ADMA have been associated with the development of pre-eclampsia. This is the first study to implicate the DDAH/ADMA pathway as a mechanism that might underlie the poor trophoblast invasion seen in this common pregnancy disorder.

**STUDY FUNDING/COMPETING INTEREST(S):**

B.A.L. was supported by a grant from Action Medical Research UK (SP4577). A.E.W. was supported by a grant from the Wellcome Trust (091550). There are no competing interests and the authors have no conflict interest to declare.

## Introduction

In normal healthy pregnancies extravillous trophoblast (EVT) cells of the placenta invade the decidua where they interact with and ultimately replace the vascular smooth muscle and endothelial cells of the maternal spiral arteries. This is a well-orchestrated process dependent on the fine balance between EVT cell proliferation and cell death mediated through apoptotic mechanisms (Smith *et al.*, 1997b; Huppertz *et al.*, 2006). Any disturbance in this balance can result in shallow EVT invasion and poor spiral artery remodelling, as seen in pre-eclampsia (PE) and fetal growth restriction (FGR) (DiFederico *et al.*, 1999; Kadyrov *et al.*, 2001). Early onset PE is a significant cause of maternal and neonatal morbidity and mortality affecting up to 5% of all births. Babies born of PE pregnancies are at increased risk of developing cardiovascular and metabolic diseases later in life (Davis *et al.*, 2012). The mechanisms responsible for the poor trophoblast invasion and inadequate remodelling have yet to be determined although a reduced capacity to invade and/or increased sensitivity to apoptotic stimuli may play a significant role.

A number of agonists trigger apoptosis, including tumour necrosis factor-α (TNFα), TNF-related apoptosis inducing ligand (TRAIL) and Fas ligand (FasL), which interact with members of the TNF death receptor family. TRAIL exists either as type II membrane bound homotrimer or as a soluble fragment (sTRAIL) produced following proteolytic cleavage. Both the membrane bound and the soluble forms of TRAIL can rapidly induce apoptosis. TRAIL interacts with five receptors, TRAIL R1 (TR1) and TRAIL R2 (TR2), also known as DR4 and DR5 respectively, two decoy receptors (DcR4 and DcR5), and a soluble receptor called osteoprotegerin (Sheridan *et al.*, 1997; Emery *et al.*, 1998). EVT express both TRAIL and its receptors, however TRAIL is also expressed by other cells at the maternal fetal interface including decidual stromal and immune cells early in gestation (Phillips *et al.*, 1999). Although EVT are relatively resistant to apoptotic stimulation, we have previously shown that inhibition of NO synthesis can increase their sensitivity to members of the TNF superfamily, including TRAIL (Dash *et al.*, 2007).

Nitric oxide (NO) is an important signalling molecule that acts in many tissues to regulate a diverse range of physiological processes, most notably: vasodilation, inflammation and immune function. NO is synthesized from l-arginine through the catalytic activity of the NO synthase (NOS) family of enzymes. NO has been implicated in many aspects of both normal pregnancies and those complicated by PE through its action as a vasodilator (Yallampalli and Garfield, 1993; Buhimschi *et al.*, 1995) and there is increased interest in NO as a possible therapeutic target for PE and FGR. We have previously demonstrated the involvement of endogenously produced NO in trophoblast motility and invasion and have found that NO is important in regulating the sensitivity of trophoblast to apoptotic stimuli (Cartwright *et al.*, 1999; Dash *et al.*, 2003a,b).

NO synthesis can be regulated in a number of ways including gene expression, enzymatic activation or changes in substrate or cofactor availability. In addition NO synthesis can be competitively inhibited by methylated analogues of arginine including asymmetric dimethylarginine (ADMA), which is formed following the hydrolysis of methylated proteins. Dimethylarginine dimethylaminohydrolase (DDAH) is an enzyme that catabolises the hydrolysis of ADMA to citrulline and dimethylamine (Leiper and Vallance, 1999). In early pregnancy the circulating concentration of ADMA falls, which could result in an increase in NO production and a decrease in vascular resistance and maternal blood pressure (Holden *et al.*, 1998). Throughout gestation the circulating concentration of ADMA rises, however, in PE pregnancies the rise is significantly greater (Holden *et al.*, 1998; Ellis *et al.*, 2001).

In this study we tested the hypothesis that changes in the expression of DDAH, a key regulator of NO synthesis, could lead to changes in the sensitivity of an EVT cell line to TRAIL-induced apoptosis.

## Materials and Methods

### Cell culture

SGHPL-4 cells, derived from primary human first trimester extravillous trophoblasts, have been used extensively as a model of extravillous trophoblasts (Choy and Manyonda, 1998; Cartwright *et al.*, 2002; Dash et al., 2003a) and in particular studies investigating the role of NO in apoptosis where they respond in a similar manner to primary extravillous trophoblasts (Dash et al., 2003a,b, 2005; Whitley *et al.*, 2007). SGHPL-4 cells were cultured in Hams F-10 media supplemented with 2 mM l-glutamine, 100 units/ml penicillin, 0.1 mg/ml streptomycin and 10% (v/v) fetal calf serum (FCS).

### Inhibition of DDAH expression using siRNA

SGHPL-4 cells were treated with Dharmacon siGENOME SMARTpool siRNA siRNA constructs as per the manufacturer's instructions (Little Chalfont UK). Specifically, SGHPL-4 cells were seeded in 6 well plates in 10% (v/v) FCS in Hams F10 containing glutamine and penicillin and streptomycin as detailed above. When confluent the medium was replaced with serum-free Hams-F10 without antibiotics and cultured overnight. Cells were then treated for 24 h with 20 nM siRNA and 4 µl Dharmafect in serum and antibiotic free media, and incubated for a further 48 h in Hams-F10 containing 10% FCS (v/v). A control treatment with a non-targeting siRNA was carried out concurrently, to account for any changes resulting from transfection alone. The siRNA sequences were as follows; DDAH1: GCAGCAAACUGUAUAUAUC/GAAGGAGGUUGACAUGAU/CAACAAAGGGCACGUCUUG/CAGAUGGGUUUGCAUUUGAA; DDAH2: GGAAAUAGGAGACGAGAAC/UGACAGAUCACCCAUAUGC/GCACUGACGUUCUCUUCAC/GAUCUGGCCAAAGCUCAAA; and non-targeting pool #2:UAAGGCUAUGAAGAGAUAC/AUGUAUUGGCCUGUAUUAG/AUGAACGU GAAUUGCUCAA/UGGUUUACAUGUCGACUAA.

### RNA isolation

RNA was isolated using Qiagen RNeasy RNA mini kit, as per the manufacturer's instructions (QIAGEN, West Sussex, UK).

### SYBR green quantitative RT–PCR analysis

RNA was reverse transcribed using Bioline cDNA synthesis kit according to manufacturer's instructions (Tetro cDNA kit, components: Tetro RTase, oligo (dT)_18_, random hexamer primers, ultra-pure dNTPs, RNase Inhibitor, BIOLINE, London, UK). 40 ng cDNA was used in duplicate samples for quantitative RT–PCR using Power SYBR green Master Mix (Applied Biosystems, Life Technologies, Carlsbad, CA, USA) as per the manufacturer's instructions using the following sequence specific primers: 18S: ACA-CGT-TCC-ACC-TCA-TCC-TC and CTT-TGC-CAT-CAC-TGC-CAT-TA, DDAH1: CCA-GTT-TAG-GCT-TAC-CAG-CA and TGC-AAT-GTA-GAA-GAG-GCA-CA, DDAH2: CAG-GGG-TAG-TAT-AGG-AAG-TAG and TAT-TGA-GGC-TCT-CTC-CCA-ACT-AC. Q-PCR was carried out using a Bio-Rad CFX96 Real-Time PCR Detection System (Bio- Rad, Hemel Hempstead, UK). The PCR amplification protocol was as follows: Step 1: 95°C for 10 min (enzyme activation), Step 2: 95°C for 15 s (denaturation), Step 3: 55°C for 60 s (data collection), Step 4: Repeat step 2 and 3 (40 cycles) and Step 5: Melt curve: 55–95°C at 0.5°C increments for 5 s each; the cycle threshold was <25. Primers were purchased from Primer Design (Southampton, UK) or Eurogentec (Southampton, UK) with primer efficiencies of >90% (DDAH1: 98%, 18S: 96%). Expression of analysed genes was normalized to RNA loading for each sample using the 18S ribosomal RNA as an internal standard and fold changes were calculated using the delta cT method; results were normalized to a calibrator sample to control for intra-assay variability.

### Western blot analysis

Cells were collected and lysed by scraping into RIPA buffer containing the protease inhibitors phenylmethylsulfonyl fluoride (1 mM), sodium orthovanadate (1 mM) and aprotinin (60 μg/ml). Proteins were resolved by SDS PAGE in 10% gels, and transferred to PVDF membranes. Membranes were subsequently blocked in 5% w/v skimmed milk powder dissolved in Tris buffered saline containing 0.1% v/v Tween-20 (Sigma-Aldrich, Dorset, UK) (TBS-T) for 1 h. Membranes were incubated overnight, at 4°C in goat anti Human DDAH1 antibody (1:1000 in 5% w/v skimmed milk powder/TBS-T) (Torondel *et al.*, 2010) and subsequently in secondary rabbit anti-goat HRP (Sigma Dorset UK) for 1 h at room temperature (1:10 000 in in 5% w/v skimmed milk powder/TBS-T). Detection was performed using ELC plus (Millipore, Watford, UK). Blots were subsequently blocked and re-probed with a mouse anti tubulin antibody (0.15 μg/ml; Abcam, Cambridge, UK, ab11323). Western blots were scanned and the integrated intensity of each band was determined using Image J (Schneider *et al.*, 2012). The Western blots shown are representative of at least three experiments and the results shown are relative to the intensity of tubulin.

### Determination of apoptosis

Cells were treated with siRNA as above or for 6 h with 10, 100 or 200 µM ADMA-2HCl (Sigma Dorset, UK). TRAIL was then added at a final concentration of 500 ng/ml (Keogh *et al.*, 2007; Agostinis *et al.*, 2012) and the cells were subject to time-lapse microscopy, as previously described (Dash *et al.*, 2003b). Apoptotic morphology was characterized by cytoplasmic shrinkage, nuclear condensation, a phase bright appearance, and formation of membrane blebbing and blistering (Whitley *et al.*, 2007). Cells were visualized throughout the experiment using an Olympus IX71 inverted phase-contrast microscope (Olympus, Japan) with a motorized stage (Prior scientific, Cambridge, UK) and cooled CCD camera (Hamamatsu Photonics, Hertfordshire, UK, model C4742-95) enclosed in a humidified chamber at constant 37°C (Solent Scientific, Segensworth, UK). Images were captured every 15 min over 24 h using Image Pro Plus software (media Cybernetics, Bethesda, USA). Two fields of view were captured at 10× magnification, at each time point, for each treatment group and 20 cells were analysed blind in each field of view. The time frame at which apoptotic morphology was first observed in 20 randomly selected cells, in each field of view was recorded.

### TRAIL receptor expression

Cell mono-layers were washed with PBS and detached with Versene (supplied as a 1× solution of 0.2 g/l ethylenediaminetetraacetic acid, Life Technologies, Carlsbad, CA, USA) at 37°C. Following centrifugation at 500×*g*, the cells were washed in PBS and re-suspended in FACS buffer (PBS, 0.5% w/v BSA, 0.1% NaN3). The Fc receptors were blocked by the incubation of 5 × 10^5^ cells per tube with 1 µg human IgG at 4°C for 30 min. For the detection and quantification of TRAIL R1 and R2, 2.5 × 10^5^ cells were incubated with antibodies either, TRAIL R1 (PE conjugated, FAB347P, 25 µg/ml) or R2 (Alexa 488 conjugated, FAB6311G, 50 µg/ml), the appropriate IgG control, mouse IgG_1_- PE conjugated (IC002P, 25 µg/ml) or mouse IgG_2B_ – Alexa 488 conjugated (IC0041G, 50 µg/ml), respectively as per the manufacturer's instruction. Compensation beads were used to reduce background interference (BD Bioscience, Oxford, UK). Data were collected on an LSR2 fluorescence activated cell sorter (BD Biosciences) and analysed using the Flowjo software package (Ashland, USA). Antibodies were all purchased from R&D systems (Minneapolis, MN, USA).

### Statistical analysis

All statistical analysis was carried out on GraphPad Prism6 (La Jolla, CA, USA). Data were compared using either a repeated measures ANOVA or paired *t*-test (parametric). Appropriate *post hoc* tests were applied and significance was taken as *P* < 0.05. Data are presented as the mean + SEM from at least three independent experiments.

## Results

### Sequence specific siRNA inhibits the expression of DDAH1 and DDAH2 in SGHPL-4 cells

**Figure 1:**
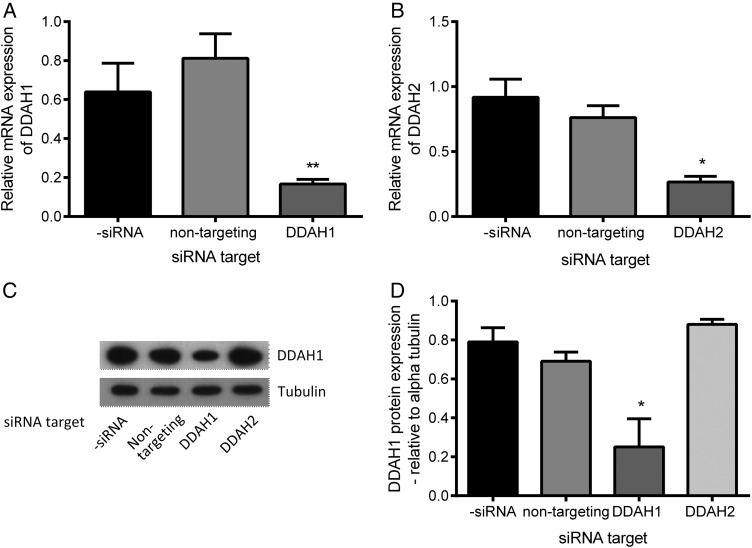
The effect of siRNA transfection on the expression of DDAH1 and 2 in SGHPL-4 cells. The effect of transfecting SGHPL-4 cells with a targeted siRNA on the expression of DDAH1 (**A**) or DDAH2 (**B**) mRNA was followed by qPCR. The specificity of the siRNA was confirmed by transfection of a non-targeting siRNA control construct. Protein knockdown of DDAH 1 by western blot analysis is demonstrated in a representative western blot for DDAH1 and Tubulin (**C**) as well as in the combined densitometric data (**D**). The data are shown as mean + SEM from at least three independent experiments, *P < 0.05, **P < 0.01 compared with non-targeting control.

To investigate the effect of DDAH1 and 2 dysregulation on SGHPL-4 apoptosis, we used siRNA to selectively inhibit expression. Following 24 h incubation with siRNA and a subsequent 48 h incubation in complete media, DDAH1 expression was significantly and specifically reduced in SGHPL-4 cells at both the mRNA and protein levels, when compared with control and the non-targeting siRNA control (Fig. 1A and B). DDAH2 mRNA expression was also specifically inhibited as detected by QRT–PCR but was not detectable by western blot analysis.

### Inhibition of DDAH1, but not DDAH2, expression increases TRAIL-mediated trophoblast apoptosis

**Figure 2:**
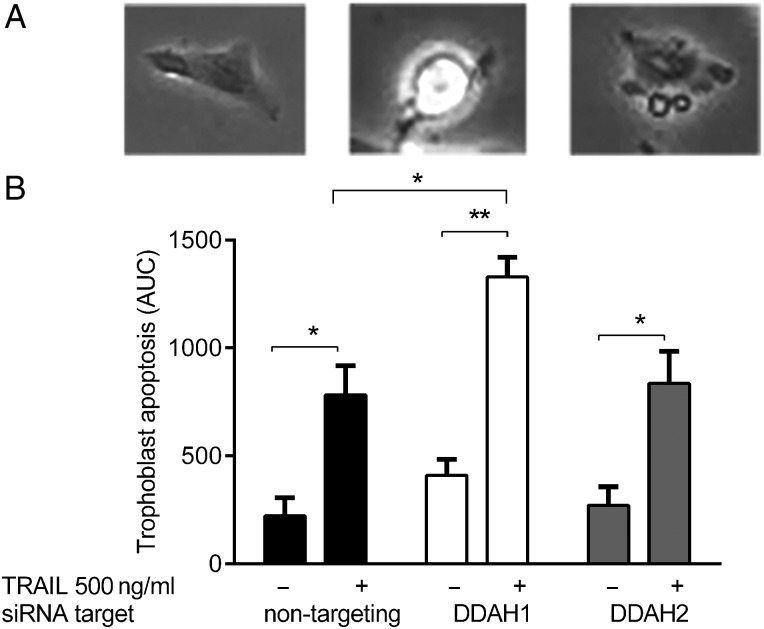
The effect of inhibiting the expression of DDAH1 and 2 on TRAIL-induced apoptosis. Time lapse apoptosis analysis was carried out on cells transfected with either non-targeting siRNA or siRNA targeted to DDAH1 or 2, following addition of 500 ng/ml TRAIL for 24 h. Twenty cells were chosen at random for each condition and induction of apoptosis was followed by characteristic morphological changes (**A**). The image on the left shows a cell prior to the onset of apoptosis, the middle image shows the same cell rounding up and becoming bright before membrane blebs and blisters develop. The image on the right shows the formation of apoptotic bodies. Kinetics curves were obtained following the induction of apoptosis and the areas under the curve (AUC) were calculated (**B**). DDAH1 knockdown resulted in a significantly increased susceptibility to TRAIL-induced apoptosis. The data are shown as mean + SEM, from at least three independent experiments, *P < 0.05, **P < 0.01.

To determine whether inhibition of DDAH expression had any effect on TRAIL-induced SGHPL-4 cell apoptosis, cells were transfected with control siRNA, or siRNA to either DDAH1 or 2. Cells were stimulated with 500 ng/ml TRAIL and the induction of apoptosis was followed by time-lapse microscopy over 24 h. There was no significant change in the sensitivity of SGHPL-4 cells in which the expression of DDAH2 mRNA was inhibited. A small (but not significant) increase in apoptosis was seen with DDAH1 inhibition even in the absence of an exogenous apoptotic stimulus. However, a significant increase in apoptosis was seen after treatment with TRAIL in the cells with inhibited DDAH1 compared with those with the siRNA control (Fig. 2).

### ADMA increases TRAIL-induced apoptosis in SGHPL-4 cells

**Figure 3:**
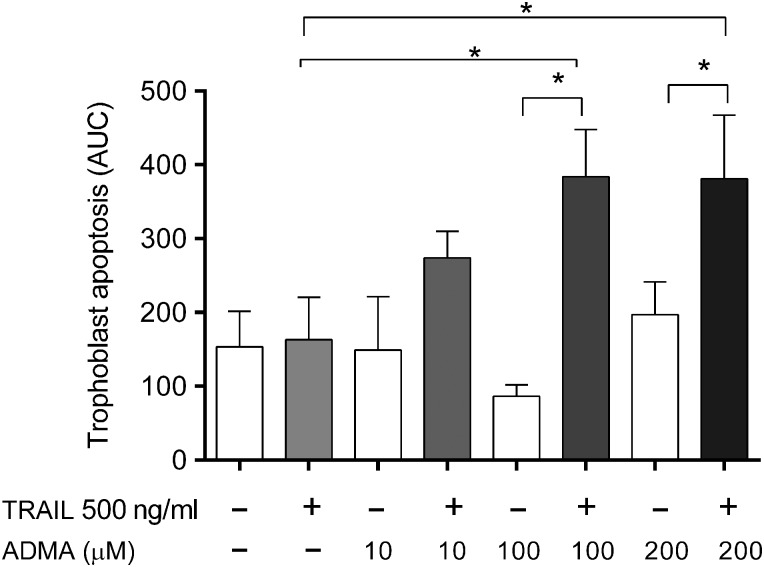
The effect of ADMA on TRAIL induced apoptosis. Untransfected SGHPL-4 cells were treated with ADMA at the doses indicated and then stimulated with 500 ng/ml TRAIL for 24 h. Apoptosis was followed by time-lapse microscopy. Adding ADMA significantly increased TRAIL-induced apoptosis of SGHPL-4 cells at both 100 and 200 µM ADMA. The data shown are the mean + SEM, from at least three independent experiments, *P < 0.05.

SGHPL-4 cells incubated with increasing concentrations of ADMA and then stimulated with 500 ng/ml TRAIL for 24 h underwent apoptosis as determined by time-lapse microscopy. A dose dependent increase in SGHPL-4 cell apoptosis was seen, reaching significance at 100 μM ADMA (*P* < 0.05) after 24 h (Fig. 3).

### Inhibition of DDAH1 increases TRAIL-mediated apoptosis by increasing TRAIL receptor 2 expression

**Figure 4:**
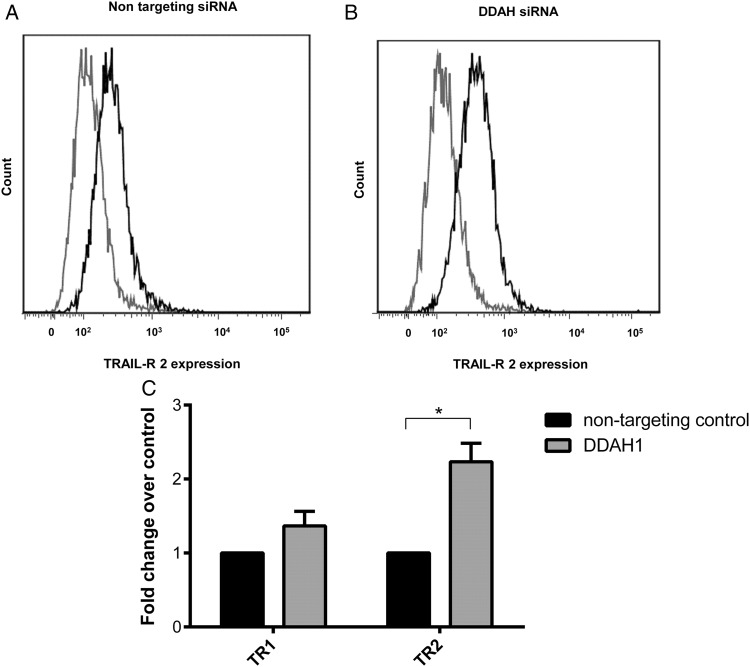
Cell surface expression of TRAIL receptor 1 and 2. The expression of TR2 by SGHPL-4 transfected with either non-targeting siRNA (**A**) or siRNA targeted to DDAH1 (**B**) was assessed using flow cytometry, the IgG control is shown in gray and the TR2 in black. TR2 is represented by the grey line and IgG control is represented by the black line. The images shown are representative of at least three independent experiments. A comparison of the effect of inhibiting DDAHI on the expression of both TR1 and TR2 is also shown (**C**). The data are shown as mean + SEM, from at least three independent experiments, *P < 0.05.

Following the finding that inhibiting DDAH1 expression increased the susceptibility to TRAIL-induced apoptosis in SGHPL-4 cells, we used FACS to look at the expression of TRAIL receptors on the surface of these cells. FACS analysis revealed a significant increase in TR2 expression in those cells treated with DDAH1 targeting siRNA compared with the non-specific siRNA (Fig. 4). Interestingly, although significant inhibition was displayed with DDAH2 targeting siRNA, these did not display the same increase in TR2 (data not shown). This could explain why TRAIL did not induce the same increase in apoptosis in these cells. TR1 did not display a similar increase in expression following either DDAH1 or 2 targeting siRNA. This may indicate a specific effect of DDAH1 on TR2 expression.

## Discussion

The key findings of the present study are: (i) SGHPL-4 cells are more sensitive to TRAIL-induced apoptosis following inhibition of DDAH1 but not DDAH2, (ii) this response could be mimicked by exogenous addition of ADMA an inhibitor of NO synthesis, and (iii) the increased sensitivity was accompanied by an increase in the expression of TR2 but not TR1.

The maternal fetal interface represents a potentially hostile environment for the invading trophoblasts not least because decidual stromal and maternal immune cells secrete a number of pro-apoptotic cytokines including TRAIL. Although the invading EVTs express the appropriate receptor repertoire, under normal circumstances they remain resistant to the apoptotic actions of these cytokines. Exaggerated trophoblast apoptosis has been associated with the poor remodelling of maternal spiral arteries seen in pregnancy complications such as PE and FGR, yet the underlying mechanisms are poorly understood (Smith *et al.*, 1997a; Leung *et al.*, 2001).

NO regulates a number of important trophoblast functions including motility and invasion (Cartwright *et al.*, 1999). Factors that regulate NO synthesis can therefore have a significant effect on establishment of a healthy pregnancy. Primary first trimester EVTs and the EVT cell line SGHPL-4 used in this study express both DDAH1 and 2 mRNA and protein (Ayling *et al.*, 2006) and this was largely supported by the findings of this study. We therefore sought to examine the role of DDAH in altering sensitivity of SGHPL-4 cells to stimulation with recombinant TRAIL by independently targeting both isoforms using siRNA. Using time-lapse microscopy, we were unable to detect a significant change in the degree of apoptosis in cells transfected with siRNA targeting DDAH2 compared with the siRNA control, however there was a significant difference in cells transfected with siRNA targeting DDAH1.

Although the two isoforms of DDAH are believed to have similar enzymatic activity, the apparent rate of ADMA metabolism for DDAH2 is almost 70 times less than that of DDAH1 (Pope *et al.*, 2009). This is consistent with our data indicating that only inhibition of DDAH1 affects trophoblast apoptosis and the growing body of evidence that DDAH2 may have different biological functions (Cooke and Ghebremariam, 2011; Hu *et al.*, 2011), as well as the demonstrated difference in tissue and cellular distribution for the two isoforms (Chen *et al.*, 2005). Complicating the picture still further is the data suggesting that both isoforms may regulate protein function via direct protein–protein interactions (Tokuo *et al.*, 2001; Hasegawa *et al.*, 2006; Leiper and Vallance, 2006). Therefore to determine whether the effect of inhibiting the expression of DDAH1 on SGHPL-4 cell sensitivity to TRAIL could be mimicked by increasing ADMA, we repeated the experiments in the presence of exogenous ADMA and found that it too, could increase the sensitivity of SGHPL-4 cells to stimulation by TRAIL. The data presented so far, together with our previous data on the regulation of trophoblast cell apoptosis, would implicate an inhibitory effect on the production of NO. We have previously shown that NO can modulate apoptosis in trophoblasts through the nitrosylation of specific cysteine residues in target proteins including protein kinase Cε and caspase 3 and 8 (Dash et al., 2003a, 2007). However a number of other mechanisms have been proposed which might regulate TRAIL-induced apoptosis, including the expression of the non-signalling decoy receptors and the redistribution of the two death receptors to and from the cell surface. EVT express both TR1 and TR2 and although activation of either receptor can lead to apoptosis, it is believed that it is activation of TR2 that is the predominant receptor subtype involved in the mediation of cell death in many cell types (Schneider-Brachert *et al.*, 2013). Inhibition of DDAH1 expression resulted in a significant increase in cell surface expression of TR2 and a small but not significant increase in the expression of TR1. A similar increase in surface expression of TR2 by trophoblasts has been reported previously in response to TNF-α (Bai *et al.*, 2009).

In conclusion, it is known that inadequate trophoblast invasion in the first trimester of pregnancy is associated with poor spiral artery remodelling and the most severe forms of PE and FGR. The mechanisms underlying this failure to invade are unclear yet an understanding at a cellular or molecular level is fundamental if we are to ameliorate or prevent these pregnancy complications. In this study, we demonstrate a DDAH1 isoform specific effect on the sensitivity of trophoblasts to stimulation by the apoptotic cytokine TRAIL. We further demonstrate, that exogenous ADMA, the substrate for DDAH also sensitizes trophoblasts to TRAIL. Finally we show that inhibition of DDAH1 expression is associated with increased expression of TR2. It is therefore possible that the increased ADMA seen in pregnancies complicated by pre-eclampsia reflects a much earlier imbalance in the DDAH/ADMA system leading to the increased trophoblast apoptosis seen in this syndrome.

## Authors' roles

B.A.L., J.E.C., G.S.W. conceived and designed all experiments and the experiments were performed by B.A.L. and A.E.W. The manuscript was prepared by B.A.L., K.L., J.E.C. and G.S.W. and all authors critically revised and approved the final manuscript.

## Funding

B.A.L. was funded by Action Medical Research (AMR SP4577) and A.E.W. was funded by the Wellcome Trust (091550). Funding to pay the Open Access publication charges for this article was provided by the Wellcome Trust.

## Conflict of interest

None declared.
